# Thorium lung burdens of NORM workers revisited

**DOI:** 10.1093/rpd/ncag035

**Published:** 2026-04-14

**Authors:** Gregory Stanley Hewson, Martin I Ralph, Marcus Cattani

**Affiliations:** School of Medical and Health Sciences, Edith Cowan University, 270 Joondalup Drive, Joondalup, Western Australia 6027, Australia; School of Medical and Health Sciences, Edith Cowan University, 270 Joondalup Drive, Joondalup, Western Australia 6027, Australia; School of Medical and Health Sciences, Edith Cowan University, 270 Joondalup Drive, Joondalup, Western Australia 6027, Australia

## Abstract

Historical thorium bioassay data collected from Australian mine workers in the 1990s, including *in vivo* lung counting and thoron-in-breath (TIB) measurements, were re-evaluated using current dosimetric and biokinetic models. Revised daily thorium intakes from bioassay were compared with previous estimates to assess changes in the dose profile and potential implications for contemporary operations. Reanalysis revealed lower estimates of annual dose, including the number of workers assessed as exceeding 20 mSv. However, it was found that intakes derived from industry personal air sampling (PAS) underestimated intake by up to three-fold. This study determined that sensitive bioassay techniques, such as TIB using an electrostatic collection chamber, are feasible for detecting low thorium lung burdens in longer-term workers at current mineral processing operations involving naturally occurring radioactive materials (NORM). This study improves the accuracy of historical exposures and highlights that PAS protocols require improvement to enhance radiation protection practices in industries handling NORM.

## Introduction

Workers involved in operating and maintaining treatment plants that process minerals containing naturally occurring radioactive materials (NORM) are potentially at risk of radiation exposure from intake of dust [1]. The internal radiation dose from the intake of dust arises primarily from alpha particle emitters of the thorium and uranium series. In the Western Australian mineral sand industry, all the valuable minerals contain enhanced levels of radioactivity, particularly the mineral monazite, an important source of rare earth elements, which contains 5%–7% thorium and 0.1%–0.3% uranium [2]. The mineral sand industry has been operating continuously for 70 years in Western Australia and has often attracted public attention and scrutiny in relation to occupational radiation exposure. In the late 1980s, there were reports of excessive committed effective internal radiation doses (hereafter referred to as dose) of >20 mSv year^−1^ being received by many mineral sand industry workers [3]. The elevated doses prompted improvements in engineering controls and the use of respiratory protection to reduce worker doses and provided a catalyst for several research studies to better define the aerosol parameters (particle size and solubility) and the retention and excretion of thorium by industry workers [1].

Since 2000, the maximum annual doses from the intake of airborne radioactivity have been maintained below 5 mSv in mineral sand treatment plants, with mean annual doses of <1 mSv [4]. However, over this period, the market for monazite was depressed and this mineral was not separated as a saleable product in mineral separation plants. With the increasing demand for rare earth elements to support the energy transition, the industry has commenced reprocessing previously stockpiled monazite-rich concentrates. This may have implications for the increased intake of airborne radioactivity by workers; hence, it is prudent to revisit past bioassay research into thorium retention and excretion, especially in the context of current dosimetric and biokinetic models [5–7].

Two studies [8, 9] measured lung retention of thorium via *in vivo* lung counting and *in vitro* thoron (^220^Rn)-in-breath (TIB) tests. The studies interpreted the significance of the results using the LUDEP software application [10], which was based on the first iteration of the International Commission on Radiological Protection (ICRP) human respiratory tract model (HRTM) [11]. The analysis at that time concluded that 32 workers (15% of those tested) were estimated to have received an average annual internal dose >20 mSv over the duration of their employment in the industry. Nearly all those estimated to be above an annual average of 20 mSv had been employed for more than 6 years (geometric mean length of employment of 12.3 years). Terry *et al.* [9] concluded that most of the inhaled radioactivity was due to intake in the early part of the worker’s employment, prior to the introduction of workplace improvements in dust control and occupational hygiene practices.

Forty-seven percent of the 209 workers tested via TIB had results below the minimum detection limit (MDL) of the equipment, which corresponded to 5 mSv year^−1^ for workers with >5 years of chronic intake. Hence, there remained a knowledge gap in relation to the likely exposure of this large cohort of workers, who had, ostensibly, similar exposure profiles to the other workers according to company inhalation intake estimates for the workers based on personal air sampling (PAS) of total alpha activity.

Notwithstanding the magnitude of the thorium lung burdens found in these studies and the benefits of individual intake monitoring, no further bioassay studies have been reported in the Western Australian mineral sand industry since the late 1990s. A key finding from earlier bioassay studies was the significant underestimation of daily intake derived from PAS measurements compared with intakes derived from bioassay measurements. The underestimation was greatest for workers with <8 years of chronic intake, raising questions about the validity of industry PAS protocols and the appropriateness of ICRP default model parameter values used in past dose calculations [9].

The publication of the ICRP’s Occupational Intakes of Radionuclides (OIR) series [5–7, 12] provides an opportunity to re-evaluate historic bioassay data and hence update past estimates of worker intakes and doses. Such re-evaluations have recently been reported for past urinary and fecal thorium excretion measurements [13, 14]. Furthermore, previously reported doses from industry PAS programs will now be higher for the same recorded intake because the inhalation dose coefficient for a default 5 μm AMAD, Type S ^232^Th aerosol in ICRP 137 [6] is 4.5 times higher than the ICRP 68 [15] value applied in the past [16].

Ensuring a scientifically robust estimate of intake is important from operational, regulatory and safety perspectives, as overestimation of intake may lead to inefficient control measures, resource allocation, and regulatory oversight, whereas underestimation of intake could increase workers’ health risk. The output from a retrospective evaluation of bioassay data should indicate the need for updates to operational radiation protection guidelines and enhance the reliability of future long-term health studies in NORM-exposed worker populations.

The objectives of this study were to: (i) re-evaluate past thorium lung burden data using current internal dosimetry models to update thorium intakes and assess the consequent impact on doses, including sensitivity to model parameters; (ii) compare revised bioassay-derived thorium intakes with historic PAS-derived intakes; and (iii) assess the feasibility of thorium lung burden measurements on the contemporary mineral sand industry workforce based on their exposure records.

## Methods

Bioassay data were collected from two studies [8, 9], each comprising *in vivo* lung counting and *in vitro* analysis of TIB, which were conducted on Western Australian mineral sand workers in the 1990s. The materials and methods used to collect and analyse the data are described in these publications and in a research report [17]. In brief, 207 workers from five mineral sand separation plant sites in Western Australia were selected by their employer to participate in TIB testing according to broad exposure criteria determined by the researchers, with an emphasis on the selection of long-term industry workers. TIB measurements were conducted at the worker’s job site using a double filter tube device described by Terry and Hewson [8, 17] and based on the design of Mayya [18]. The gross alpha count from ^212^Bi and ^212^Po (i.e. ^220^Rn progeny) collected on the second (exit) filter was converted to the activity of ^224^Ra freely emanating ^220^Rn at the mouth.

*In vivo* thorium lung burdens for 19 of the 207 workers were assessed using the whole-body monitoring facility at the Australian Radiation Laboratory (now the Australian Radiation Protection and Nuclear Safety Agency) in Melbourne. These workers were selected based on their TIB results, employment duration, and recorded exposure history. It was necessary to select workers with anticipated significant thorium lung burdens because of the relatively high MDL for *in vivo* lung counting (~10–15 Bq). TIB measurements from the same group of 19 workers were used to determine the average thoron exhalation rate as a percentage of the lung burden. A mean exhalation rate of 3.7 ± 1.5% was derived from these comparative measurements, and this factor was used to convert the measured ^224^Ra emanating at the mouth from the TIB test to a thorium lung burden. The individual *in vivo*- and TIB-derived lung burden measurements used in this research were extracted from the two published studies [8, 9] and the research report [17].

Daily inhalation of thorium over each worker’s employment period (time elapsed from commencement in industry to bioassay measurement date) was estimated in the original studies from company employment records, annual radiation reports submitted to the government regulatory agency, and a retrospective assessment of thorium intake pre-1986 [19]. These estimates were based on PAS measurements of both long-lived alpha activity and gravimetric dust concentrations extracted from archived research records. No changes were made to the historical PAS-derived intake estimates previously assigned to each worker.

Analysis of the TIB data was previously undertaken using the LUDEP software application [10] to obtain the relationship between lung retention of thorium and chronic intake (Bq day^−1^) of 10 μm AMAD, Type S thorium ore dust as a function of intake duration. The use of a 10 μm AMAD for inhaled dust was based on personal cascade impactor measurements taken from mineral sand workers during the 1990s [20].

An analysis of the combined dataset from the two bioassay studies had not been done previously, and this study re-analysed the complete dataset using the prospective calculation function of the Taurus internal dosimetry software application (version 2.2, 2024) [21] based on the latest ICRP publications [5, 6]. To ensure consistency with previous analyses, similar assumptions were made in relation to chronic intake of thorium ore dust, aerosol AMAD, and lung solubility, except if noted otherwise. Table 1 summarizes the intake assumptions and measurement details used in the two earlier studies.

**Table 1:** Intake assumptions and measurement details from earlier thorium lung burden studies on Western Australian mineral sands workers.

Parameter/item	Value/description
No. mineral sands sites	5—denoted Site A, B, C, D, and E
Aerosol AMAD (GSD)	10 μm (2.5)
Aerosol solubility	Type S
Mode of breathing^a^	0.85 nose, 0.15 mouth
Intake	Chronic inhalation^b^
Internal dosimetry software	LUDEP
Intake estimate (^232^Th.day^−1^)	≤1985: retrospective assessment >1985: industry PAS records
^232^Th inhalation dose coefficient^c^	0.054 mSv.Bq^−1^ (0.056 mSv.Bq^−1^)
Thoron-in-breath device (*n* = 207)	Double filter tube (MDL = 0.27–0.40 Bq of emanating ^224^Ra)
*In vivo* lung counting (*n* = 19)	2-4x NaI(Tl) detectors (150 mm x 75 mm), 2x LEGe detectors (MDL ~ 10–15 Bq ^228^Ac)

^a^In this study the LUDEP generated retention curve for a nose breathing worker was used to allow direct comparison with the Taurus curve under a similar assumption.

^b^The intake is assumed to be averaged over the employment period (i.e. time from start of intake to bioassay measurement date) and does not account for the cyclic nature of intake.

^c^Dose coefficient derived from LUDEP for the thorium series in equilibrium based on 85% nose breathing and 15% mouth breathing. For a nose breathing worker doing heavy work the LUDEP-derived coefficient was 0.056 mSv.Bq^−1^.

Microsoft Excel was used to download and analyse data from Taurus and produce thorium lung retention curves as a function of chronic intake duration. Polynomial functions were fitted to the retention curves to estimate a worker’s daily ^232^Th intake by comparing the measured thorium lung burden with the predicted retention at 1 Bq day^−1^ for the exposure duration at the time of measurement. Additionally, Taurus software was used to investigate other aerosol parameters to determine sensitivity of dose estimates to model inputs.

Inhalation dose coefficients for the thorium series were determined utilizing the nuclide mixture features of the Taurus software. A total intake of 5 Bq day^−1^ was used for a thorium series mixture, with 20% activity allocated to the first five radionuclides in the series, ^232^Th, ^228^Ra, ^228^Ac, ^228^Th, and ^224^Ra, to represent secular equilibrium (i.e. 1 Bq day^−1^ for each radionuclide).

Amendments were made to the TIB-derived thorium lung burdens for the 62 workers reported in the first study [8] as a result of refinements arising from the second study [9] as follows:

- The thoron exhalation factor was updated based on 13 additional *in vivo* lung counting measurements (i.e. 3.7% vs. 4.7% reported in Study 1).
- An experimentally determined calibration factor for the TIB unit of 3.35E-03 (to convert the measured alpha particle count to ^224^Ra activity) was used instead of the theoretical factor (3.05E-03) applied in Study 1.

Hence, for this analysis the TIB-derived thorium lung burdens reported in Study 1 were increased by a factor of 1.4 (i.e. [0.047/0.037] × [0.00335/0.00305]).

Studies of *in vivo* and *in vitro* thorium lung burdens for other thorium dust exposed workers were obtained from a literature search to compare the results from those studies with the Western Australian mineral sand worker data.

## Results

### Revised estimated internal doses from *in vivo* lung counting

**Figure 1:**
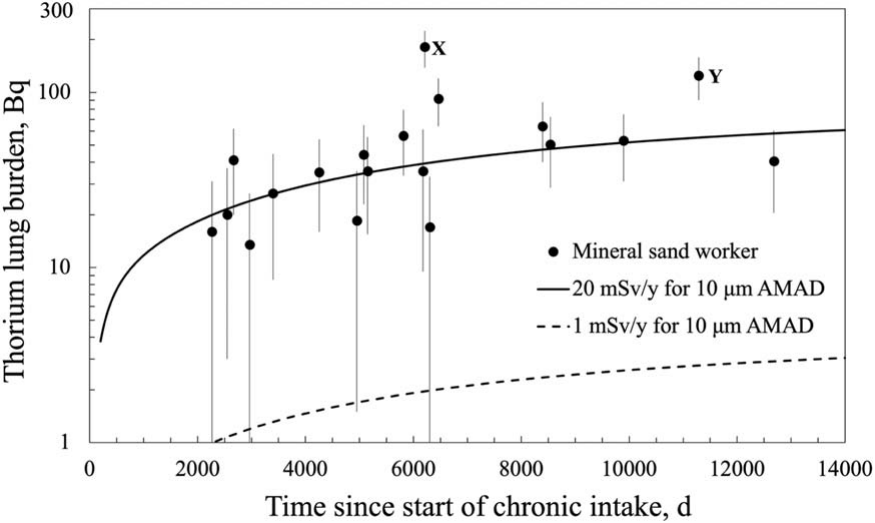
Thorium lung burdens of mineral sand industry workers (n = 19) assessed by *in vivo* lung counting. Error bars are 95% confidence levels.

The combined data from previous *in vivo* lung counting measurements are shown in Fig. 1. The Taurus generated retention curve (solid line) in Fig. 1 corresponds to an average intake of 1 Bq day^−1^ of 10 μm AMAD, Type S ^232^Th for a nose-breathing worker doing heavy work, and an annual dose of 20 mSv for the thorium series, assuming secular equilibrium within the dust matrix (i.e. 1 Bq day^−1^ × 365 days × 0.054 mSv.Bq^−1^). The dashed line corresponds to 0.05 Bq day^−1^ and an annual dose of 1 mSv.

Based on the 20 mSv year^−1^ curve, 11 of the 19 workers tested were estimated to have received an average annual dose of 20 mSv or more over their intake period. Worker X recorded the highest lung burden measured (181 Bq), corresponding to an average annual dose of 90 mSv and an estimated cumulative dose over 17 years of 1530 mSv at the time of testing. Worker Y was estimated to have a cumulative dose of 1340 mSv after working for 31 years in the industry.

### Revised estimated internal doses from thoron-in-breath measurements

**Figure 2:**
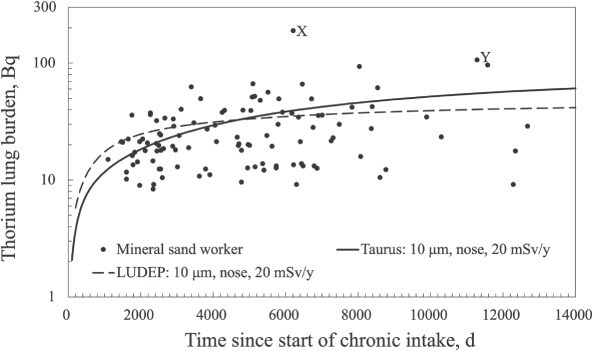
Derived thorium lung burdens for mineral sand industry workers (*n* = 110) from TIB measurements plotted against time since start of chronic intake. The Taurus and LUDEP bioassay retention curves are based on inhalation of 1 Bq day^−1^, Type S ^232^Th by nose-breathing male workers (refer text).

The combined data from the two TIB studies are shown in Fig. 2, together with two lung burden curves: one generated from a previous LUDEP evaluation, and the other generated from Taurus. Both curves are based on intake data shown in Table 1, and are related to the inhalation of 1 Bq day^−1^ of Type S ^232^Th. Both the Taurus and LUDEP curves for a nose-breathing worker corresponded to an annual internal dose of ~20 mSv. For graph clarity, the TIB data points for the 97 workers with results below the detection level (thorium lung burden of ~7–11 Bq) have not been plotted.

The Taurus lung burden curve for a nose-breathing worker shows less thorium lung retention than the LUDEP curve for chronic intake periods of <10 years (3650 days) but greater lung retention after 17 years (6200 days).

The best-fit regression line for the Taurus and LUDEP lung burden curves shown in Fig. 2 was used to convert the worker’s TIB-derived thorium lung burden measurement to the estimated daily ^232^Th intake. The two curves for 1 Bq day^−1^ intake of 10 μm AMAD, Type S ^232^Th by a nose-breathing male worker, and breathing at 1.7 m^3^ h^−1^ (heavy work) are characterized by lognormal and polynomial functions as per the following equations: 


(1) \begin{eqnarray*}&& \mathrm{LUDEP}\ \mathrm{curve}:\mathrm{Th}\ \mathrm{lung}\ \mathrm{burden}\ \mathrm{at}\ 1\ \mathrm{Bq}\ {\mathrm{day}}^{-1}= 8.994\nonumber\\&& \quad \times \ln \left(\mathrm{d}\right)\hbox{-} 43.938\ \left({\mathrm{r}}^2=0.99\right) \end{eqnarray*}

(2) \begin{eqnarray*}&& \mathrm{Taurus}\ \mathrm{curve}:\mathrm{Th}\ \mathrm{lung}\ \mathrm{burden}\ \mathrm{at}\ 1\ \mathrm{Bq}\ {\mathrm{day}}^{-1} \nonumber\\&& \quad = {9.70 \mathrm{E} {-} 19} \times{\mathrm{d}}^5-3.71\mathrm{E}{-}14\times{\mathrm{d}}^4+5.18\mathrm{E}\nonumber\\&& \quad{-}10\times{\mathrm{d}}^3 -3.36\mathrm{E}{-}06\times{\mathrm{d}}^2+1.46\mathrm{E}{-}02\nonumber\\&& \quad \times \mathrm{d}\ \left(\mathrm{intercept}=0,{\mathrm{r}}^2=0.999\right) \end{eqnarray*}

Where the intake period, d, is in days.

Hence, for the worker denoted ‘X’ in Fig. 2, with a measured thorium lung burden of 190 Bq after 6209 days (17 years) of chronic intake, his average daily thorium intake over his employment duration was estimated at 5.3 and 4.7 Bq day^−1^ from LUDEP (Equation 1) and Taurus (Equation 2), respectively. For the worker denoted ‘Y’ the difference in average daily intake was more pronounced between LUDEP (3.1 Bq day^−1^) and Taurus (2.2 Bq day^−1^) due to this worker’s 31 years intake period.

Note: The parameters for the best-fit polynomial equation vary slightly, depending on the duration of chronic intake. In this study, an intake duration of 14 000 days (~38 years) was used because the longest employment duration of the workers tested was 35 years.

**Figure 3:**
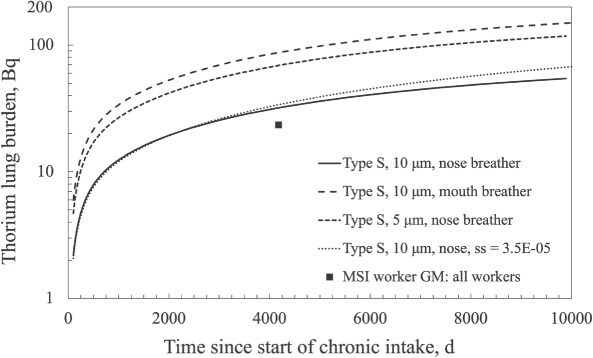
Thorium retention curves at 1 Bq day^−1^ intake as a function of various model input parameters. The geometric mean (GM) result for 110 mineral sand industry (MSI) workers also shown.

The TIB-derived average yearly dose over the worker’s intake period (i.e. years elapsed from the start of employment to the time of the TIB test) was determined by the product of the average yearly intake of ^232^Th (365.25 x daily intake) and the applicable dose conversion factor for the thorium series obtained from Taurus. Table 2 summarizes the geometric mean doses across all workers greater than the MDL and various intake parameter assumptions. Figure 3 shows the Taurus generated retention curves for the intake scenarios listed in Table 2.

**Table 2:** Variations in geometric mean daily ^232^Th intakes and annual doses derived from TIB data as a function of biokinetic model, mode of breathing and aerosol AMAD.^a^

Parameter	Unit	Value (GSD)
No. tested >MDL of TIB method	-	110
TIB: GM intake period	year	11.4 (1.8)
TIB: GM ^232^Th lung burden	Bq	23.3 (1.8)
PAS: GM ^232^Th daily intake	Bq day^−1^	0.54 (2.1)
PAS: annual dose (ICRP-OIR updated)	mSv year^−1^	10.6 (2.1)
TIB: LUDEP: nose breather^b^ (@ 0.76 Bq day^−1^)	mSv year^−1^	15.6 (1.8)
TIB: Taurus: nose breather^c^ (@ 0.77 Bq day^−1^)	mSv year^−1^	15.1 (1.8)
TIB: Taurus: mouth breather^c^ (@ 0.27 Bq day^−1^)	mSv year^−1^	12.3 (1.9)
TIB: Taurus: nose breather^c^, 5 μm AMAD (@ 0.34 Bq day^−1^)	mSv year^−1^	12.3 (1.9)
TIB: Taurus: nose breather^c^, s_s_ = 3.5E-05 day^−1^ (@ 0.68 Bq day^−1^)	mSv year^−1^	18.0 (1.9)

^a^All dose coefficients used relate to inhalation of Type S thorium series aerosol with an AMAD of 10 μm and the default slow absorption rate, unless stated otherwise.

^b^The LUDEP derived dose coefficient is 0.056 mSv Bq^−1^.

^c^The Taurus derived dose coefficients, in mSv Bq^−1^, for males doing heavy work are as follows:

• nose breather with 5 and 10 μm AMAD: 0.100 and 0.054, respectively

• mouth breather with 10 μm AMAD: 0.128

• nose breather with 10 μm AMAD and a slow absorption rate (s_s_) of 3.5E-05: 0.072.

The analysis in Table 2 shows a similar estimated geometric mean annual dose for a nose-breathing worker between LUDEP (original assessment) and Taurus (revised assessment) using a 10 μm AMAD as the base case. It can be seen from Fig. 2 that at the geometric mean intake period of 11.4 years (4200 days), both retention curves are similar. The inhalation dose coefficients for the thorium series were similar (0.056 vs. 0.054 mSv Bq^−1^).

With the current ICRP OIR model, the assumption of nose breathing and the use of a 10 μm AMAD for mineral sand workers gives a higher annual dose result compared to using a 5 μm AMAD or assuming mouth breathing. The dose coefficients for a mouth-breathing worker, inhaling a dust distribution with a 10 μm AMAD, or a nose-breathing worker inhaling dust with a 5 μm AMAD, are approximately two-fold higher than the dose coefficient for a nose-breathing worker inhaling dust with a 10 μm AMAD. However, the daily ^232^Th intake derived from the Taurus retention curves for the 10 μm AMAD mouth breather and 5 μm AMAD nose breather scenarios were approximately two-fold lower, thus countering the impact of the higher dose coefficient, resulting in similar annual dose estimates for the same measured thorium lung burden. A higher annual dose is estimated if the slow absorption rate (designated as s_s_ in the ICRP lung model) is reduced by approximately one-third from 1E-04 to 3.5E-05 day^−1^. This value was chosen based on a recent analysis by Hewson *et al.* [16].

Table 3 summarizes the dose profile obtained from LUDEP and Taurus for intake of a 10 μm AMAD, Type S aerosol, together with the PAS estimates of the dose for the 110 workers above the MDL of the TIB technique. The first PAS estimate (ICRP 68) is based on the approved intake-to-dose protocol applied at the time of the bioassay studies [22]. The second PAS estimate is based on applying the updated dose coefficient for the thorium series as per ICRP OIR [12].

**Table 3:** Estimated internal doses to 110 workers based on TIB and PAS measurements, based on application of LUDEP and Taurus.

Dose range	Number of workers			
	LUDEP^a^ (previous)	Taurus^b^ (current)	PAS_past_^b^: ICRP 68	PAS_updated_^c^: ICRP-OIR
<5 mSv	1	4	45	20
5– < 10 mSv	28	24	42	26
10– < 15 mSv	27	24	7	28
15– < 20 mSv	17	20	14	13
20– < 30 mSv	23	23	2	8
30– < 50 mSv	11	14	-	15
>50 mSv	3	1	-	-

^a^The retention curves for a nose breather as shown in Fig. 2 were used to estimate the dose for each worker based on the worker’s daily intake derived from bioassay.

^b^Based on historical daily ^232^Th intake derived from PAS. A dose coefficient of 0.029 mSv Bq^−1^ was previously used based on ICRP 68 [15] values for 10 μm AMAD thorium ore dust (as per regulatory approval at the time).

^c^Based on historical daily ^232^Th intakes derived from PAS and re-analysed using the updated ICRP OIR [12] dose coefficient of 0.054 mSv Bq^−1^ for 10 μm AMAD thorium ore dust.

The updated assessment indicated that 38 workers were estimated to receive an annual dose of more than 20 mSv, and only one worker was estimated to have an average annual dose of more than 50 mSv over their employment period. Previously (via LUDEP), 37 and 3 workers received annual doses of more than 20 mSv and 50 mSv, respectively. Hence, the dose profile distribution was similar to that estimated previously.

The PAS distribution of doses provides a substantially different result and is representative of what was recorded in the worker dose records at that time. Using the original approved dose protocol, the annual doses calculated from PAS intake indicated only two workers above 20 mSv, with most workers (*n* = 87) in the testing cohort assessed as below 10 mSv. The retrospective dose assessment from the PAS-derived intakes using the updated dose coefficient showed better alignment with the bioassay-derived doses; however, PAS still underestimated the dose compared to TIB measurements.

### Lung burden estimates—personal air sampling vs. thoron-in-breath

**Figure 4:**
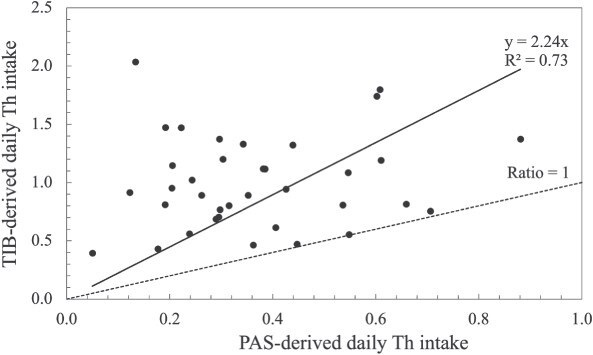
Comparison of daily thorium intake derived from TIB and PAS for workers (*n* = 36) with intake period <8 years.

TIB study 2 [9] compared the ratio of predicted (via TIB) to measured (via PAS) daily thorium intake and reported a range of ratios from 0.43 to 13.5 with a mean ratio of 2.5 ± 2.4, assuming inhalation of a 10 μm AMAD aerosol. In this study, we compared the TIB- and PAS-derived daily intake of thorium in a subset of workers (*n* = 36) with a chronic intake period of <8 years. This subset of workers had recorded intakes based on PAS for the duration of their employment, whereas intakes for longer term workers (>8 years) were less reliable because retrospective assessments were required. Figure 4 shows a comparative analysis using combined data from the two TIB studies. The data are relatively scattered; however, all data points except one have a TIB:PAS ratio > 1. Least squares regression analysis returned a slope of 2.24 (r^2^ = 0.73). Significant inter-worker variability was noted for workers with similar PAS-derived daily intakes, and the range of TIB to PAS ratios was 1.0 to 15 with a mean ratio of 3.6 ± 2.8 for a 10 μm AMAD aerosol.

### Literature values for thorium lung burden measurements

Summaries of the literature values from published bioassay studies are provided in Tables 4 and 5 for *in vivo* lung counting and TIB testing, respectively. Only studies since 1985 were included, as earlier studies were less useful because of the relatively high detection limits associated with the analytical methods and equipment available at the time. These tables update those previously reported [8].

**Table 4:** Literature values of *in vivo* thorium lung burdens for thorium dust-exposed workers.

Exposure situation, country	Exposure period (year)	No. of tests	Thorium lung burdens (Bq)^a^		Ref.
			Mean	Range	
Monazite treatment—Brazil^b^	17–25	2	131	107–155	[23]
Thorium refinery—India	10–32	5	28^*^	15–67	[24]
Thorium refinery—Brazil	14–28	7	24	<10–44	[25]
Thorium gas mantle—Brazil^c^	?	38	<10	<10–34	[26]
Mineral sands—Australia^d^	2–40	19	51	11–181	[9]

^a^Geometric mean indicated by ^*^.

^b^Values for ^228^Ac. ^208^Tl was found to be 27% lower in one worker and 38% lower in the other worker, indicating disruption to thorium series equilibrium in the lung.

^c^Based on 38 tests of ^228^Ac across six workers. Only 11 tests were above the MDL of 10 Bq. The authors concluded that worker exposure to elevated levels of ^220^Rn progeny in the workplace led to higher *in vivo* counts for ^212^Pb, ^212^Bi, and ^208^Tl.

^d^Average of ^228^Ac and ^208^Tl peaks.

**Table 5:** Literature values of TIB measurements for thorium dust-exposed workers.

Exposure situation, country	Exposure period (year)	No. of tests	Emanating ^224^Ra at mouth (Bq)		Ref.
			Mean^a^	Range	
Thorium refinery^b^, India	<12–>25	176	1.2	<0.6–19	[18]
Rare-earth & iron mine, China	0.5–27	130	0.05	<0.04–0.55	[27]
Thorium refinery, India	5–30	10	1.5	0.93–2.8	[28]
Thorium gas mantle factory, India	25	2	3.9	1.4–6.3	[28]
Mineral sands^c^, Australia	1.4–40	207	1.1	<0.31–7.0	[8, 9]
Gas mantle plant, Germany	0.5–22	14	0.27	<0.05–0.85	[29]
Rare-earth & iron mine^d^, China	8–36	64	0.17	0.013–0.73	[30]
Rare-earth refinery, China	1.6 ave.	77	0.12	<0.04–0.33	[31]
Mineral sands, India^e^	1–30	196	3.0	0.76–8.6	[32]

^a^Arithmetic means.

^b^Maximum value obtained after a weekend break from employment was 4.2 Bq.

^c^Reworked combined data from two TIB studies as per this paper. Mean based on 110 workers above the MDL.

^d^Data were collected from December 1993 to August 1994. The results refer to dust-exposed workers in the crushing area of the refinery. Converted from tabulated lung burden results based on an assumed thoron exhalation rate of 10%. Note: TIB measurements at the Chinese rare-earth and iron mine were undertaken on 638 thorium dust-exposed workers between 1983 and 1994, with mean and maximum ^224^Ra values of 0.16 and 1.11, respectively [33].

^e^Converted from tabulated lung burden results based on a thoron exhalation rate of 9% as used by the authors.

## Discussion

Follow-up bioassay studies on mineral sand workers tested in the past should improve knowledge of the extent of clearance of thorium from the lungs. However, it is anticipated that most of the workers who participated in bioassay studies in the early- to mid-1990s would have left the industry or retired, since >25 years have now elapsed. At the time of testing, 60% of workers tested had already been employed for more than 10 years. Nevertheless, the feasibility of performing follow-up tests should be investigated.

The elevated dose estimates obtained from previous *in vivo* lung counting and *in vitro* TIB studies (as indicated in Table 3) were not included in the worker’s dose records maintained by the worker’s employer, and the findings did not result in changes to operational or regulatory radiation protection practices. Two principal reasons for this include: (i) the studies being deemed research, and bioassay not being a regulator approved dose assessment method at the time; and (ii) revised dose coefficients published in ICRP 68 [15] were adopted by the local regulatory agency towards the end of the study period (i.e. late 1990s). The revised dose coefficient for the thorium series was 3.5-fold lower than the previous ICRP 30 dose coefficient [16]; hence, doses estimated from PAS were reduced by 3.5-fold based on the same intake. This change has a consequential impact on the need for further improvements and research, including bioassay studies [16].

### Implications of updated models and impact on dose estimates

Reanalysis of the *in vivo* measurements confirmed that long-term workers involved in the operation and maintenance of mineral sand separation plants incurred a significant intake of thorium. The estimated doses from the *in vivo* measurements were not reported in the two previous studies [8, 9] and it is significant that two workers, with 17 and 31 years of employment, had accumulated estimated lifetime doses (from intake alone) of more than 1000 mSv. The employment duration of the 11 workers assessed as being ≥20 mSv year^−1^ from the *in vivo* tests ranged from 7 to 31 years; hence, many of these workers had worked in the mineral separation plants during the 1970s and/or the 1980s when occupational hygiene practices, particularly the control of dusty processes, were less robust [2]. It is likely that mineral sand workers not involved in the bioassay studies but with similar long-term employment experience would have also accumulated substantial lung burdens of thorium and lifetime doses.

A comparison of the distribution of TIB-derived doses between LUDEP and Taurus indicated a similar estimated annual mean dose across all workers who tested above the MDL from 15.6 to 15.1 mSv (Table 2). This outcome was anticipated given the shapes of the two retention curves shown in Fig. 2. In earlier studies, the LUDEP-derived dose coefficient (0.056 mSv Bq^−1^) was different from that derived from ICRP 68 data (0.029) because a slow absorption class was assumed for all radionuclides in the thorium series when applying the LUDEP model. Overall, the re-evaluation of TIB-derived doses in this study essentially confirmed the robustness of the dosimetry analysis conducted in earlier studies, with minor adjustments.

The finding that PAS-derived estimates of dose were much lower than those indicated by the bioassay studies (Table 3, PAS: ICRP68) was not explicitly stated in earlier research, and hence no changes were made to the regulatory approved dose protocols based on PAS. The lack of change likely resulted in the systemic under-reporting of PAS-derived doses from thorium intake from 1997 until the introduction of updated ICRP OIR dose coefficients into the prescribed legislative framework in 2019 [34]. The potential for the recorded PAS-derived intakes and doses to be underestimated over this 22-year period should be considered in future health-effect studies. The updated PAS-derived doses (Table 3, PAS: ICRP-OIR), while better aligned with the bioassay-derived data, continued to highlight the potential underestimation of the dose. This is unlikely to be due to an incorrect assumption of the solubility class for mineral sand dust but may indicate the use of an inappropriate AMAD for the collected dust, together with dust sampler bias.

### Personal air sampling vs. bioassay estimates of intake

The TIB discrepancy with PAS (Fig. 4) is consistent with the recent re-evaluation of historical urine and fecal bioassay data from mineral sand workers [13, 14], which also showed bioassay measurements indicating a significantly higher average daily thorium intake than PAS. The extent of underestimation by PAS may be greater than that indicated by this study, as no account was taken for the use of respiratory protective equipment, which was most commonly used from the late 1980s for specified dusty tasks [35].

Figure 4 shows the largest apparent underestimation of daily thorium intake occurring at PAS intakes <0.4 Bq day^−1^. This finding suggests an issue with industry PAS protocols, including the validity of worker assignments to similar exposure groups. PAS protocols used in Western Australia NORM industries have remained essentially unchanged for the last 30 years, and hence it may be reasonably concluded that similar underestimation of PAS intake is still occurring for current thorium-exposed workers.

Hewson *et al.* [36] explored the issue of PAS-derived intakes for NORM dust exposure in detail and highlighted that underestimation may arise due to sampler collection efficiency bias where coarse aerosols are present, non-accounting for sampler wall losses during radiometric analysis, incorrect breathing rate assumption, and use of the geometric mean rather than arithmetic mean concentration in the intake calculation. Alpha particle self-absorption from dust collected on PAS filters may have also been a factor in the past when dust concentrations were much higher.

ICRP highlights the poor correlation observed between PAS and bioassay measurements for both chronic and acute intake and indicates a preference for bioassays to assess individual intake [5]. However, PAS is a cost-effective approach for routine monitoring and is used extensively to assess non-radioactive airborne contaminants in NORM industries. The issues associated with current PAS protocols may be partly alleviated by using a different sampling device with a collection efficiency that more closely matches the ISO inhalability convention [37] and applying a breathing rate consistent with the tasks being undertaken.

### Comparative analysis with literature values from other studies

Tables 4 and 5 show that there was significant thorium bioassay research activity in the late 1980s and throughout the 1990s investigating the lung retention of thorium by workers in various countries and across various industrial activities. Relatively few bioassay studies have been published since this time related to the intake of thorium ore dusts [1]. A literature review revealed that no further *in vivo* thorium lung burden studies of active NORM-exposed workers have been published since the last mineral sands worker study by Terry *et al.* [9]. This is most likely due to either a reduction in thorium exposure, as has occurred in the Western Australian mineral sand industry, the cessation of monazite processing and thorium refining, or a lack of support for further research.

**Figure 5:**
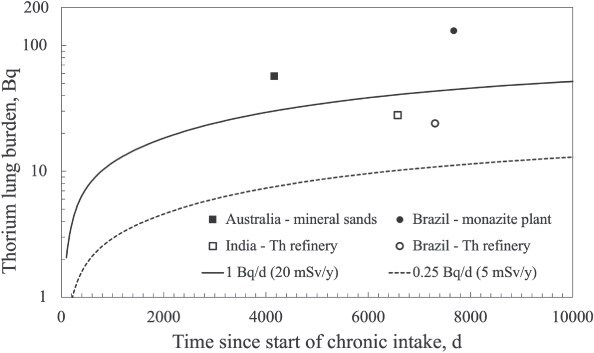
Reported mean *in vivo* measured thorium lung burdens from various thorium exposed workers. Values refer to historical *in vivo* measurements between 1989–97 (refer Table 4).

Figure 5 shows the mean thorium lung burdens measured in different groups of thorium-exposed workers, as shown in Table 4. The mean *in vivo* burden for the worker group was plotted against the mean employment time at the time of measurement. The bioassay retention curves are as per those shown in Fig. 1 and relate to the intake of a 10 μm AMAD aerosol of Type S by a nose-breathing worker. The *in vivo* thorium lung burdens for long-term workers in various NORM-related industries elsewhere have shown burdens of similar order to the Western Australian workers, with maximum burdens of ~100 Bq or more (refer Table 4). The mean lung burden implied that significant annual doses were received in all studies highlighted. However, it should be recognized that the *in vivo* measurements are highly biased because only long-term workers with significant intake are likely to have thorium lung burdens above the detection limit for lung counting.

The TIB measurements (Table 5) were not as consistent across the different industries, which is likely due to differences in the chemical and physical form of the thorium intake and thoron exhalation rate from the lung, which is dependent on the degree of translocation of radium from the lung to other organs. The most recent data from a mineral sand operation in India [32] showed TIB values three times higher than the Western Australian data; however, that study used an assumed thoron exhalation rate of 9% compared to the measured 3.7% rate used in the Western Australian studies. Hence, the range of estimated thorium lung burdens is similar for both Indian and Western Australian worker groups if the same exhalation rate is used. The inferred mean thorium lung burden for >10-year workers in the Indian study [32] was 35 Bq (with a mean employment of 18.7 years).

A significant constraint on further TIB measurements will be the assignment of an appropriate thoron exhalation rate, given that experimentally determined rates from *in vivo* lung counting of workers is now unlikely owing to relatively low intake levels in contemporary operations processing or handling NORM. Future TIB studies of Western Australian mineral sand workers can use the mean exhalation rate determined in the past; however, such a rate may not be applicable to thorium-exposed workers from other industries.

### Feasibility for bioassay monitoring of contemporary industry workers

Earlier TIB studies were performed with a double-filter tube device where the MDL equates to ~7–11 Bq of thorium lung burden, and hence only significant burdens could be detected arising from intake over many years. Later studies using an electrostatic collection chamber (ECC) achieved an MDL equivalent to ~3 Bq lung burden of thorium with a potential of 1 Bq using alpha particle vacuum spectroscopy [29, 38]. Given the downward trend in airborne thorium concentrations in the Western Australia mineral sand industry over the last 25 years [39], future studies will need to use an ECC or another sensitive measurement technique.

The mean annual internal doses for current mineral sand workers have been reported (based on PAS) to be of the order of 1 mSv [4]. Figure 1 shows that at this level of annual dose, 1 Bq in the lung is only reached after ~6 years of intake. Considering the uncertainty of PAS estimates and the likelihood that the most exposed group of workers have actual intakes twice the mean value, the intake period for a measurement >MDL reduces to 3 years. Thus, any future TIB studies will likely only be feasible on more highly exposed mineral sand workers with at least 3 years of intake.

*In vivo* detection limits for thorium in the lung (as measured by ^228^Ac or ^208^Tl) appear to still be ~10–15 Bq [6], which implies that significant chronic intake needs to have occurred to accumulate such a burden. The use of *in vivo* lung counting is not feasible for contemporary workers given that anticipated lung burdens of >15 Bq are unlikely at the currently reported daily intake of ^232^Th. Workers would have to have incurred annual internal doses in excess of 20 mSv (i.e. 365 Bq year^−1^ of ^232^Th) for several years or in excess of 10 mSv for decades (as inferred from Fig. 1) before accumulating a measurable lung burden. However, *in vivo* counting may be useful for the follow-up of past long-term workers to assess the clearance rate of thorium from the lungs.

### Further research and broader implications

Epidemiological research on radiation-induced lung disease and cancer risk relies on accurate historical exposure data. The re-evaluation and updating of radionuclide intakes from past bioassay measurements using current models can improve the worker dose assessments used in such research. The substantial reduction in the inhalation dose coefficient for the intake of thorium ore dust arising from the ICRP OIR recommendations flags the need for the retrospective amendment of past worker dose records. In addition, this study has further highlighted potential issues with the PAS protocols used by industry, and further research is required to seek improvements in PAS-derived intakes. Such research should include a review of sampling strategies, sampler aerosol collection efficiencies, radiometric (alpha particle counting) analysis protocols, and the use of realistic workplace-specific parameters in dosimetric models, including material-specific absorption rates to blood.

**Figure 6:**
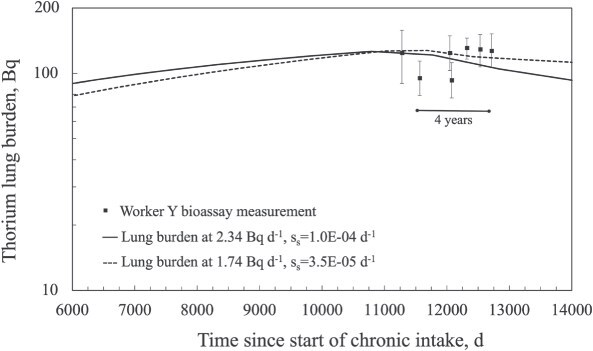
Measured thorium lung burdens for worker Y who was removed from dust exposure after ~11 500 days of chronic intake. The two curves are based on the daily intake of Type S, 10 μm AMAD ^232^Th estimated from the model fit for the inhalation scenarios shown.

In the case of inhalation of thorium associated with monazite dust, there appears to be more avid retention of thorium in the lung than that indicated by the ICRP HRTM model [5]. Terry [38] reported that subsequent TIB measurements on worker Y following removal from dust exposure showed no reduction in thorium lung burden over a 4-year period. Worker Y’s TIB data are shown in Fig. 6, together with two predicted retention curves generated from Taurus. The uncertainty of each TIB measurement was represented by a scattering factor of 1.2, based on reproducibility studies on selected workers over consecutive days in February 1996 [38]. The base case (Type S, 10 μm AMAD and default absorption parameters) does not provide a good model fit to the TIB data because the model-predicted thorium lung burden decreases more rapidly following cessation of intake compared with the measured rate. Decreasing the slow absorption rate to the blood (s_s_) by one-third improves the model fit; however, the predicted lung burdens are still below those measured. This finding indicates that further refinement may be needed for the transport rates from the alveolar (ALV) and interstitial (INT) regions of the lung model when applied to highly insoluble mineral matrices. Note that both the retention curves in Fig. 6 predict a lifetime dose of 1500 mSv for worker Y.

PAS is acknowledged as a preferred technique by regulators and industry owing to its practicality and widespread use; however, it presents a dilemma. Regulators should consider an adjustment factor applied to PAS data to account for the potential underestimation of intake. Individual monitoring for thorium retention (e.g. via TIB) may only be feasible for a select cohort of long-term workers; however, a preliminary study seems warranted, given the issues identified with PAS. Individual monitoring also allows the assessment of inter-worker variability in intake and the impact of individual work and hygiene practices.

This study revealed that two long-term workers in the past were estimated to have accumulated doses in excess of 1000 mSv, above the ICRP recommended lifetime limit, and it is likely that others who worked for similar periods had accumulated doses of a similar order. This raises the question of potential moral, legal, and financial consequences following the retrospective revision of past dose records.

The methodology used in this study can be applied to past and future bioassay studies from other industries handling NORM and thus could inform revised dose assessments and radiation protection strategies for those industries.

## Conclusions

This study re-evaluated historical thorium lung burden measurements using current ICRP biokinetic and dosimetric models. Bioassay-derived estimates of the dose were similar to those obtained from past estimates using previous models with some adjustments. However, historical PAS-derived estimates of dose, as reported in statutory records, were substantially different due to a combination of likely underestimation of intake and the use of a much lower dose coefficient, raising concerns about the accuracy of historical exposure data based on PAS. Bioassay re-evaluation confirmed that insoluble thorium, as encountered in many minerals containing NORM, is ardently retained in the lungs and continues to accumulate over the long term. The observed underestimation of intake derived from PAS compared to bioassay data highlights the need for more refined protocols to convert intake to dose, particularly in cases where workplace exposure conditions and worker practices are highly variable. This may require regulators to approve the use of a correction factor applied to historical PAS data to appropriately reflect worker intake. These findings suggest that retrospective evaluations of bioassay data incorporating the latest dosimetric approaches can enhance the accuracy of exposure assessments and consequently improve radiation protection practices. Future research should focus on improving PAS protocols, optimizing monitoring strategies, and using sensitive bioassay techniques to assess thorium retention and clearance.
